# Supplementary material for: The Potential Role of bZIP55/65 in Nitrogen Uptake and Utilization in Cucumber Is Revealed via bZIP Gene Family Characterization
Source: Plants (Basel). 2023 Sep 11;12(18):3228. doi: 10.3390/plants12183228 (PMC10537890; doi:10.3390/plants12183228)
Supplement: Supplementary file 1 [file plants-12-03228-s001.zip › Supplementary Figures S1-S3.pdf]

plants

MDPI

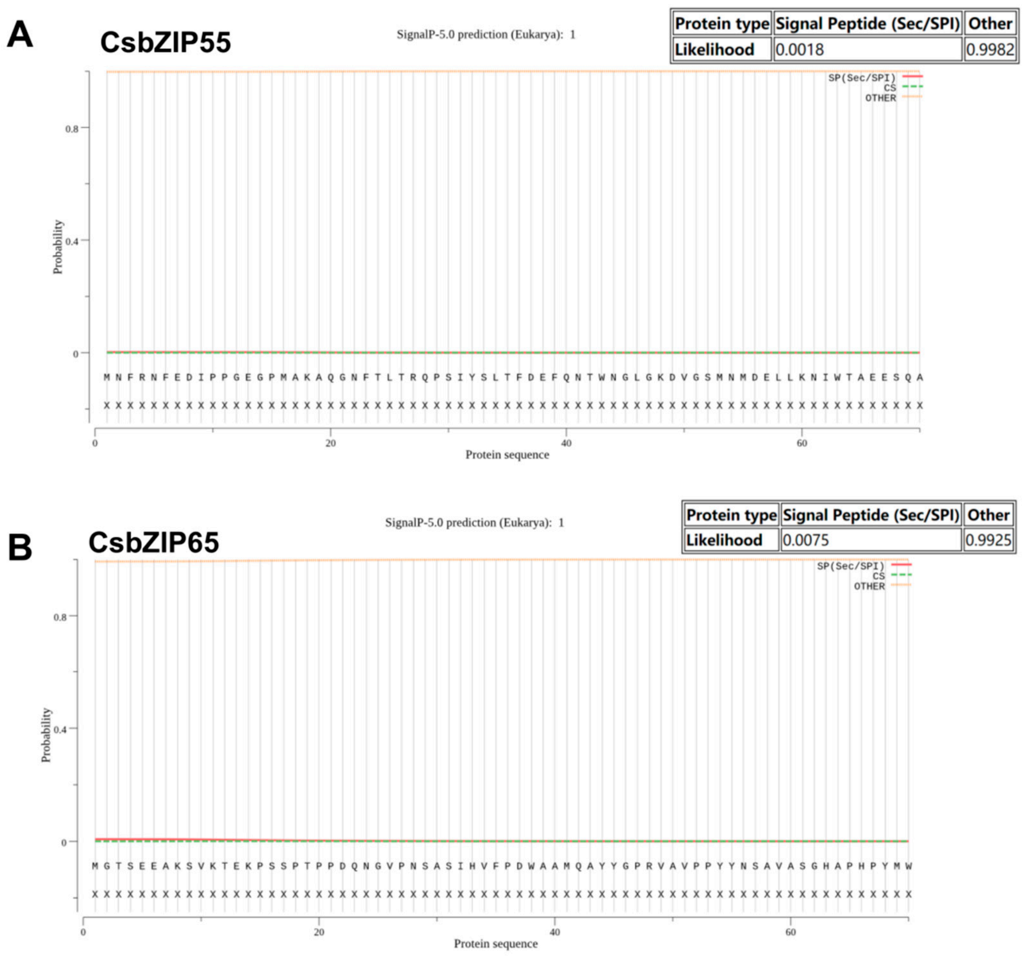

Supplementary Figure S1. Signal peptide prediction of CsbZIP55 and CsbZIP65 using SignalIP5.0.

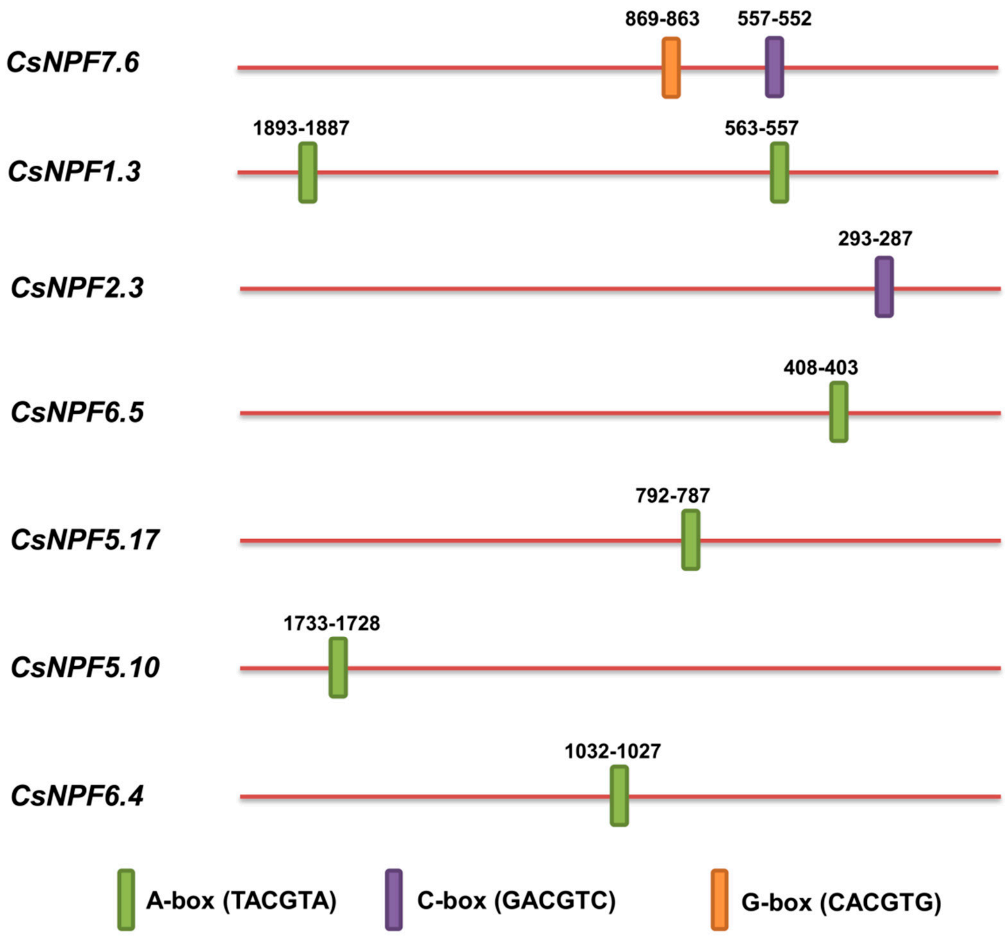

Supplementary Figure S2. The specific binding site of bZIP transcription factors in the promoter region of CsNPF genes. The green boxes, purple boxes, and orange boxes show the A-box (TACGTA), C-box (GACGTC), and G-box (CACGTG), respectively.

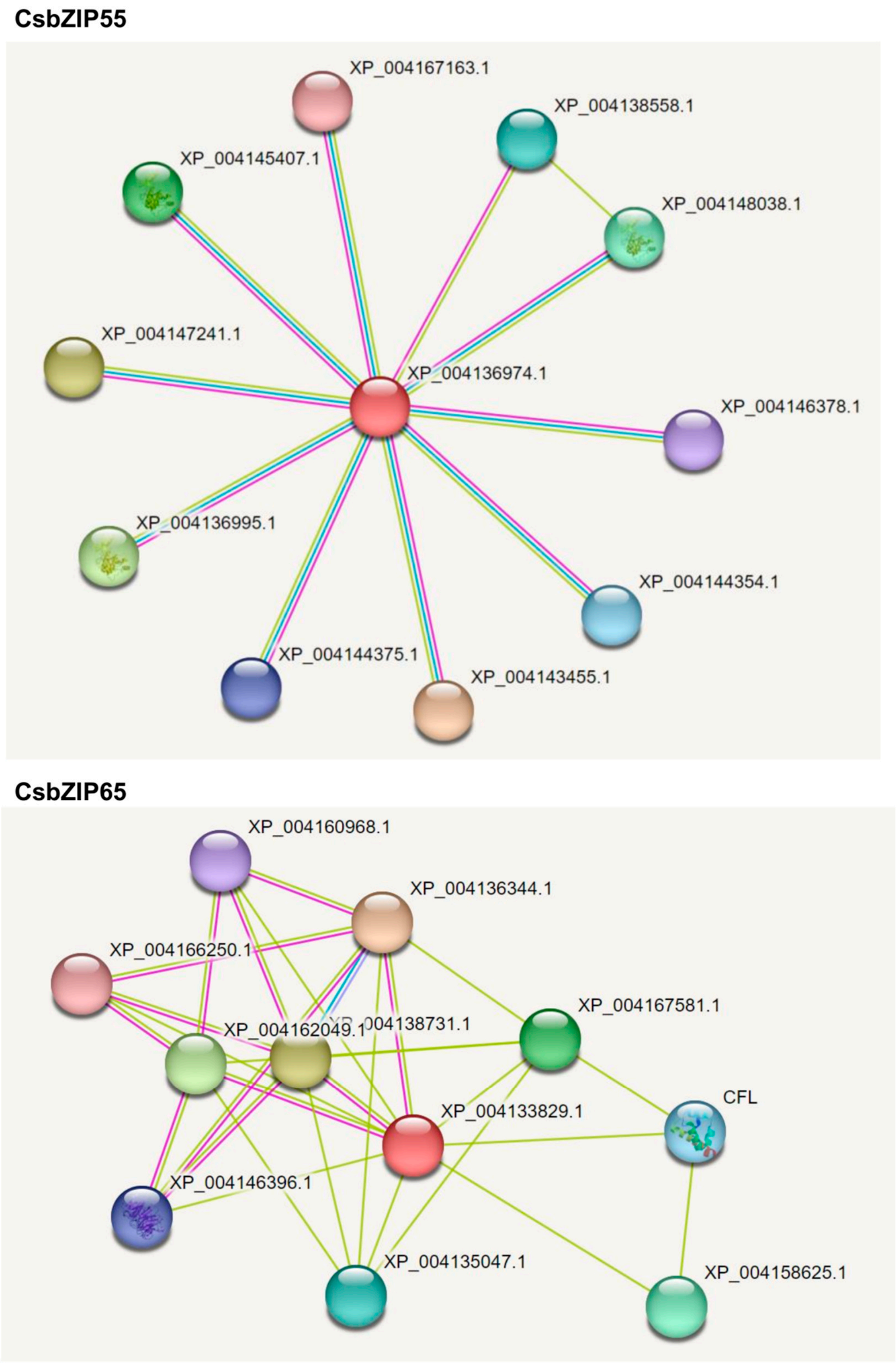

Supplementary Figure S3. Putative proteins interacting with CsbZIP55 and CsbZIP65 in cucumber. Putative proteins predicted by STRING.
